# Modulus of continuity of controlled Loewner–Kufarev equations and random matrices

**DOI:** 10.1007/s13324-020-00366-3

**Published:** 2020-05-08

**Authors:** Takafumi Amaba, Roland Friedrich

**Affiliations:** 1 Fukuoka University, 8-19-1 Nanakuma, Jônan-ku, Fukuoka, 814-0180 Japan; 2 Department of Mathematics, Saarland University, 66123 Saarbrücken, Germany

**Keywords:** Loewner–Kufarev equation, Grassmannian, Witt algebra, Faber polynomial, Grunsky coefficient, Signature, Control function, Primary 93C20, Secondary 30F10, 35C10, 58J65

## Abstract

First we introduce the two tau-functions which appeared either as the $\tau$-function of the integrable hierarchy governing the Riemann mapping of Jordan curves or in conformal field theory and the universal Grassmannian. Then we discuss various aspects of their interrelation. Subsequently, we establish a novel connection between free probability, growth models and integrable systems, in particular for second order freeness, and summarise it in a dictionary. This extends the previous link between conformal maps and large *N*-matrix integrals to (higher) order free probability. Within this context of dynamically evolving contours, we determine a class of driving functions for controlled Loewner–Kufarev equations, which enables us to give a continuity estimate for the solution of such an equation when embedded into the Segal–Wilson Grassmannian.

## Introduction

The class of univalent functions is an extraordinarily rich mathematical object within the field of complex variables, with deep and surprising connections with, e.g. conformal field theory (CFT), random matrix theory and integrable systems, cf. [6–9, 15, 19, 21, 23], just to name the most important ones in our context. So, for ${\mathbb {D}} := \{ z \in {\mathbb {C}} | |z|<1 \}$, the open unit disc, with boundary the unit circle, i.e. $S^1 = \partial {\mathbb {D}}$, let

$$\begin{aligned} {\mathcal {S}}_{{\text {reg}}} := \{ f: {\mathbb {D}} \rightarrow {\mathbb {C}}~|~\text { univalent and regular up to }S^1, f(0)=0 \text { and }f'(0)=1 \}, \end{aligned}$$

be the class of schlicht functions. Then, for every $f \in {\mathcal {S}}_{{\text {reg}}}$, $D := f( {\mathbb {D}} )$ is a simply connected domain containing the origin, with boundary $C := \partial D$, a Jordan contour. If $\hat{{\mathbb {C}}} := {\mathbb {C}} \cup \{ \infty \}$ is the Riemann sphere, let $D^c := \hat{{\mathbb {C}}} \setminus D$ be the complement of *D* in the extended complex plane.

The set ${\mathcal {C}}$ of all such Jordan contours encircling the origin forms an infinite dimensional manifold [10, 19].

It has been shown by Kirillov and Juriev [9] that there exists a canonical bijection

$$\begin{aligned} {\mathcal {S}}_{{\text {reg}}} \cong {\text {Diff}}_+ (S^1) / S^1 \end{aligned}$$

which endows ${\text {Diff}}_+(S^1)/S^1$ with the structure of an infinite-dimensional complex manifold.

Geometrically, $\pi : {\mathcal {C}} \rightarrow {\mathcal {S}}_{{\text {reg}}}$ is a fibre bundle, with fibre ${\mathbb {R}}_+^*$, which is a consequence of the Riemann mapping theorem. There exist two continua of global sections $\sigma _{r_i} : {\mathcal {S}}_{{\text {reg}}} \rightarrow {\mathcal {C}}$, $r_i>0$, $i=1,2$, such that the leaves ${\mathcal {C}}_{r_i} := \sigma _{r_i} ({\mathcal {S}}_{{\text {reg}}})$ stratify ${\mathcal {C}}$, i.e. ${\mathcal {C}} = \biguplus _{r_{1}>0} {\mathcal {C}}_{r_{1}} = \biguplus _{r_{2}>0} \widetilde{{\mathcal {C}}}_{r_{2}}$, either according to the conformal radius $r_{1} > 0$, or alternatively, the interior area $r_{2}>0$, as in [19].

Krichever, Marshakov, Mineev-Weintstein, Wiegmann and Zabrodin [10, 14, 23], motivated by the Hele-Shaw problem, cf. [15] and the monograph by Gustafsson‘et al. [5], introduced a new set of co-ordinates, the so-called harmonic moments of the interior and exterior domain, with respect to a family of harmonic functions. Namely, for $\{ z^{-k} \}_{ k \in {\mathbb {Z}}_{\geqslant 0} }$ the interior harmonic moments are given by:

1.1 $$\begin{aligned} t_k := - \frac{1}{\pi k} \int _{D^c} z^{-k} \text {d}^2 z = \quad \oint _{\partial D} z^{-k} {\bar{z}} \text {d}z, \end{aligned}$$

where the second equality is a consequence of Stokes’ Theorem. Further,

$$\begin{aligned} t_0 := \frac{1}{\pi } \int _D \text {d}^2 z. \end{aligned}$$

is the area with respect to Lebesgue measure. The set

$$\begin{aligned} \mathbf{t }_{\pm } := ( t_0, t_1, {\bar{t}}_1 , t_2, {\bar{t}}_2 , t_3, {\bar{t}}_3 , \dots ) \in {\mathbb {R}}_+ \times {\mathbb {C}}^{{\mathbb {N}}}, \end{aligned}$$

with ${\bar{t}}_k$ denoting the complex conjugate of $t_k$, are local co-ordinates on the manifold (moduli space) of smooth closed contours ${\mathcal {C}}$, cf. [19].

The other set of (natural) co-ordinates is given by the coefficients of the normalised Riemann mapping. So, we have two different sets of co-ordinates for ${\mathcal {C}}$, as shown below:

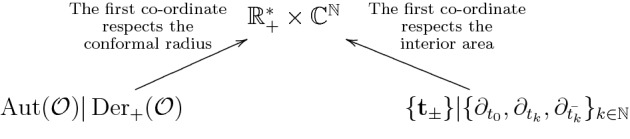



Hence, the tangent space to ${\mathcal {C}}$ permits also two descriptions, namely, as [4, 7, 10, 19]

$$\begin{aligned} {\text {Der}}_0 ( {\mathcal {O}} ) := z {\mathbb {C}} [\![ z ]\!] \partial _z \quad \text {and} \quad \{ \partial _{t_0}, \partial _{t_k}, \partial _{\bar{t_k}} \}_{k\in {\mathbb {N}}} \end{aligned}$$

Define $\ell _n := -z^{n+1} \frac{d}{dz}$, $n \in {\mathbb {N}}$, which span the positive part of the Witt algebra, i.e. for $n,m\in {\mathbb {Z}}$

$$\begin{aligned}{}[ \ell _m, \ell _n ] = (m-n) \ell _{m+n}. \end{aligned}$$

The $\partial _{t_k}$ can be determined either by specific boundary variations of the domain, which do only change one harmonic moment at the time, cf. [10, Formula (2.11)], or in terms of the Faber polynomials [19]. Combining results in [10] with our considerations, the relation for the different tangent vectors is given by

### Proposition 1.1

The vector fields $\{ \partial _{t_{k}} \}_{k \geqslant 1}$ on ${\mathcal {C}}$ and the operators $\{ \ell _{k} \}_{k \geqslant 1}$ are related by

$$\begin{aligned} \partial _{t_{k}} t_{l} = \frac{1}{ k \pi } \oint _{ \partial D^{c} } \xi ^{-l} \delta n ( \xi ) \vert \text {d}\xi \vert = \delta _{kl}, \end{aligned}$$

where

$$\begin{aligned} \delta n ( \xi ) := \partial _{n} \frac{-1}{2 \pi i} \oint _{\infty } ( \ell _{k} G_{0} )( z, \xi ) \frac{ \text {d}z }{z}, \end{aligned}$$

and $\partial _{n}$ is the normal derivative on the boundary $\partial D^{c}$ with respect to $\xi \in \partial D^{c}$, and $G_{0}( x, \xi )$ is the Dirichlet Green function associated to the Dirichlet problem in $D^{c}$.

Krichever, Marshakov, Mineev-Weinstein, Wiegmann and Zabrodin [8, 14, 23], in different constellations, defined the logarithm of a $\tau$-*function* which for a contour $C = \partial D$ is given by [8, 19]

$$\begin{aligned} \ln ( \tau ): {\mathcal {C}} \ni C \mapsto - \frac{ 1 }{ \pi ^{2} } \int _{D} \int _{D} \ln \Big \vert \frac{1}{z} - \frac{1}{w} \Big \vert \text {d}^{2}z \text {d}^{2}w \in {\mathbb {R}}. \end{aligned}$$

The $\tau$-function connects complex analysis with the dispersionless hierarchies and integrable systems [8].

A key result, which expresses the Riemann mapping in terms of the $\tau$-function, is the following

### Theorem 1.2

([8, 19]) Let $g : \hat{{\mathbb {C}}} \setminus D \rightarrow \hat{{\mathbb {C}}} \setminus {\mathbb {D}}$ be the conformal map, normalised by $g( \infty ) = \infty$ and $g^{\prime } ( \infty ) > 0$. Then the following formula holds:

$$\begin{aligned} \ln ( g(z) ) = \ln (z) - \frac{1}{2} \frac{\partial ^2\ln (\tau )}{\partial t^2_0} - \sum _{k=1}^{\infty } \frac{z^{-k}}{k} \frac{\partial ^2 \ln (\tau )}{\partial t_0\partial t_n}. \end{aligned}$$

This formula would be a key to describe the solution to the conformal welding problem (c.f. [22]) associated to Malliavin’s canonic diffusion [13] within the framework of Loewner–Kufarev equation, which would be a future work.

Another interpretation of the $\tau$-function, given by Takhtajan [19, Corollary 3.10], is that it is a Kähler potential of a Hermitian metric on $\widetilde{{\mathcal {C}}_a}$, $a>0$.

Kirillov and Juriev [9], defined a two-parameter family (*h*, *c*) of Kähler potentials $K_{h,c}$ on the determinant line bundle ${\text {Det}}^*$ over the (Sato)–Segal–Wilson Grassmannian, where *h* is the highest-weight and *c* the central charge of the CFT. For $h=0$ and $c=1$, i.e. the free Boson, one has [6, 9] for the metric

$$\begin{aligned} g_{0,1}(f) = \text {e}^{{-K_{0, 1}(f)}} \text {d} \lambda \text {d} {\bar{\lambda }} \end{aligned}$$

where $\lambda$ is the co-ordinate in the fibre over the schlicht function *f*. In terms of the Grunsky matrix $Z_f$, associated to an $f \in {\mathcal {S}}_{{\text {reg}}}$, the Kähler potential for $h = 0$, $c = 1$ is given by

$$\begin{aligned} K_{0,1}(f) =\ln \det (1-Z_f \bar{Z_f}). \end{aligned}$$

The following diagram summarises our discussion so-far:

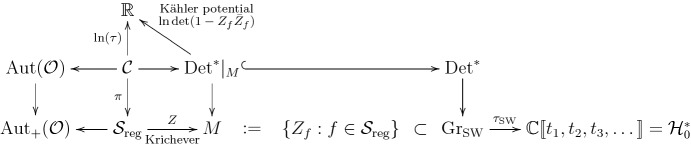



where *Z* is the Grunsky matrix, cf. [9] and ${\mathcal {H}}^*_0$ the charge 0 sector of the boson Fock space [7, p.279]. For the second square from the left, we should note that the Krichever mapping does not distinguish ${\mathcal {S}}_{{\text {reg}}}$ and ${\mathcal {C}}$ algebro-geometrically. Namely, the Krichever embedding of a Riemann mapping uses only the negative part of the Grunsky coefficients $b_{-m,-n}$, $m,n =1, 2,\dots$ but not $b_{0,0}$. One finds from the defining equation of the Grunsky coefficients that $b_{0,0}$ is the only entry of the Grunsky matrix which depends on the conformal radius. Consequently, Krichver’s embedding forgets about the conformal radius. But in order to keep track of the modulus of the derivative of the normalised Riemann mapping, we put that information into the determinant line bundle. This is the mapping ${\mathcal {C}} \rightarrow \text {Det}^{*}\vert _{M}$.

The structure of the rest of the paper is as follows: In Sect. 2 we establish a relation between the theory of Laplacian growth models, and their integrable structure, with a class of random matrices and second order free probability. We succinctly summarise it in a dictionary. In Sect. 3 we consider controlled Loewner–Kufarev equations and recall the necessary facts. Then, in Sect. 4, we give several estimates for the Grunsky coefficients associated to solutions to a controlled Loewner–Kufarev equation. Proofs of several estimates which need results from [1] are relegated to Appendix A. Finally, we prove Theorem 3.3 in Sect. 5.

## Integrability and higher order free probability

Another motivation in the works of Marshakov et al. was the close connection random matrix theory has with (Laplacian) growth models and integrable hierarchies. Takebe, Teo and Marshakov discussed the geometric meaning of the eigenvalue distribution in the large *N* limit of normal random matrices in conjunction with the one variable reduction via the Loewner equation [18]. In [1] we established and discussed a relation between CFT and free probability theory. Here we briefly present a novel connection between integrable hierarchies, large *N* limits of Gaussian random matrices and second (higher) order free probability [3]. First, consider, cf. [19, p. 42],

$$\begin{aligned} \langle \!\langle j(z) j(w) \rangle \!\rangle \end{aligned}$$

the normalised current two-point functions for free Bososn on $\hat{{\mathbb {C}}}\setminus D$, and the analogous correlation function with the Dirichlet boundary conditions, i.e.

$$\begin{aligned} \langle \!\langle j(z) j(w) \rangle \!\rangle _{\text {DBC}}. \end{aligned}$$

Further, let, cf. [3, p. 11],

$$\begin{aligned} G(z,w):=\frac{M(\frac{1}{z},\frac{1}{w})}{zw}, \end{aligned}$$

be the second order Cauchy transform, and $M(\frac{1}{z},\frac{1}{w})$ the second order moments. We obtain

### Theorem 2.1

Assume that the second order free cumulants *R*(*z*, *w*) vanish, i.e. we have an integrability / zero-curvature condition, such as for e.g. the Gaussian and Wishart random matrices. Then the tensor corresponding to the second order Cauchy transform $G(z,w) \text {d} z \otimes \text {d} w$ is given by the Ward identity

2.1 $$\begin{aligned} G(z,w) \text {d}z \otimes \text {d}w= & {} \langle \!\langle j(z) j(w) \rangle \!\rangle - \langle \!\langle j(z) j(w) \rangle \!\rangle _{\text {DBC}} \end{aligned}$$

2.2 $$\begin{aligned}= & {} \left( \frac{ G^{\prime }(z) G^{\prime }(w) }{ ( G(z) - G(w) )^2 } - \frac{1}{(z-w)^2} \right) \text {d}z \otimes \text {d}w \end{aligned}$$

2.3 $$\begin{aligned}= & {} \sum _{m,n=1}^{\infty }z^{-m-1}w^{-n-1}\frac{\partial ^2\ln (\tau )}{\partial t_m\partial t_n}\text {d}z\otimes \text {d}w \end{aligned}$$

where *G*(*z*) is the first order Cauchy transform.

This suggests us to make a dictionary translating the language from integrable systems and free probability. Table 1 is an attempt to list objects in these fields sharing the same algebraic relations.

**Table 1:** Dictionary between Laplacian growth models and free probability theory

Laplacian Growth Model (dToda hierachy)	Free Probability Theory
Exterior of the domain $D, D^{c}$	$\text {Spectral measure}^c$
Harmonic moments of *D*, $v_{n} = \frac{1}{2\pi i} \int _{\partial D} z^{n} {\overline{z}} \text {d}z = \frac{\partial \log \tau }{\partial t_{n}},$ $v_{0} = \frac{2}{\pi } \int _{D} ( \log \vert z \vert ) {\overline{z}} \text {d}z = \frac{\partial \log \tau }{\partial t_{0}}$ [19, Corollary 3.3]	?
Derivative of harmonic moments, $\frac{\partial v_{m}}{\partial t_{n}} \quad \text {for }m,n \geqslant 1$	The second order limit moments, $\alpha _{m,n} \quad \text {for }m,n \geqslant 1$
Riemann mapping, $G: D^{c} \rightarrow {\mathbb {D}}^{c}$	The first order Cauchy transform, $G(x) = \frac{M(\frac{1}{x})}{x}$
Normalised reduced 2-point current correlation function of free bosons on ${\mathbb {P}}^{1}$ parametrised by $C = \partial D$, $$\begin{array}{l} G(z,w) = \langle \!\langle \jmath (z) \jmath (w) \rangle \!\rangle - \langle \!\langle \jmath (z) \jmath (w) \rangle \!\rangle _{\text {DBC}}\\ = \frac{\partial ^{2} G_{0}(z,w)}{\partial z \partial w} - \frac{\partial ^{2}G_{\text {DBC}} (z,w)}{\partial z \partial w}\\ = \sum _{m,n=1}^{\infty } z^{-m-1} w^{-n-1} \frac{ \partial ^{2} \ln ( \tau ) }{ \partial t_{m} \partial t_{n} },\end{array}$$ where $G_{0} (z,w)$ is the Green function associated with the ${\overline{\partial }}$-Laplacian $\triangle _{0} = - \frac{1}{2} * {\overline{\partial }} * {\overline{\partial }}$ and $G_{\text {DBC}}$ is the Green function associated to $\triangle _{0}$ on $D^{c}$ [19, Theorem 3.9]	The second order Cauchy transform, $G(x,y) = \frac{M(\frac{1}{x}, \frac{1}{y})}{xy}$
Normalised 1-point current correlation function of free bosons on $\mathbb {CP}^{1}$ parametrised by $C = \partial D$, $\langle \!\langle \jmath (z) \rangle \!\rangle = - \sum _{n=1}^{\infty } z^{-n-1} \frac{ \partial \log \tau }{ \partial t_{n} }$ [19, Theorem 3.1]	?

From the above it follows now, that general higher order free (local) cumulants are given by Ward identities of *n*-point functions of the twisted Boson field over arbitrary Riemann surfaces.

## Controlled Loewner–Kufarev equations

The connection with the Loewner equation, the class of schlicht functions and integrable systems was established by Takebe et al. [18]. They showed that both the chordal and the radial Loewner equation give consistency conditions of such integrable hierarchies. A particularly important class of such consistency conditions can be obtained from specific control functions.

In the previous paper [1], the authors introduced the notion of a solution to the *controlled Loewner–Kufarev equation* (see [1, Definition 2.1])

3.1 $$\begin{aligned} \text {d} f_{t}(z) = z f_{t}^{\prime } (z) \{ \text {d} x_{0} (t) + \text {d} \xi ( \mathbf{x }, z )_{t} \}, \quad f_{0} (z) \equiv z \in {\mathbb {D}} \end{aligned}$$

where ${\mathbb {D}}= \{ \vert z \vert < 1 \}$ is the unit disc in the complex plane, $x_{0} : [0,T] \rightarrow {\mathbb {R}}$,

$$\begin{aligned} x_{1}, x_{2}, \ldots : [0,T] \rightarrow {\mathbb {C}} \end{aligned}$$

are given continuous functions of bounded variation, called the *driving functions*. We define

$$\begin{aligned} \xi ( \mathbf{x }, z )_{t} := \sum _{n=1}^{\infty } x_{n}(t) z^{n}. \end{aligned}$$

In the current paper, we determine a class of driving functions for which we establish the continuity of the solution, as a curve embedded in the (Sato)–Segal–Wilson Grassmannian, with respect to time. For this reason, we introduce the following class of controlled Loewner–Kufarev equations.

Let us first recall from the monograph by Lyons and Qian [12, Sect. 2.2] a few basic notions. For a fixed $T \geqslant 0$, let $\Delta _T:\{(s,t):0\leqslant s \leqslant t\leqslant T\}$ be the two-simplex.

### Definition 3.1

(*see* [12, Sect. 2.2]) A continuous function

$$\begin{aligned} \omega : \{ (s,t) : 0 \leqslant s \leqslant t < +\infty \} \rightarrow {\mathbb {R}}_+, \end{aligned}$$

is called a *control function* if it satisfies super-additivity:

$$\begin{aligned} \omega (s,u) + \omega (u,t) \leqslant \omega (s,t), \end{aligned}$$

for all $0 \leqslant s \leqslant u \leqslant t$, and vanishes on the diagonal, i.e. $\omega (t,t)=0$, for all $t\in [0,T]$.

Now, let *V* be a Banach space and $X: [0,T] \rightarrow V$, $T \geqslant 0$, be a path such that

$$\begin{aligned} \vert X_t - X_s \vert \leqslant \omega (s,t), \quad 0 \leqslant s \leqslant t \leqslant T, \end{aligned}$$

for a control function $\omega : \Delta _T \rightarrow {\mathbb {R}}_+$. Then *X* is called a Lipschitz path controlled by $\omega$.

We have the following

### Definition 3.2

Let $\omega$ be a control function. The driven Loewner–Kufarev equation^1^ (3.1) is *controlled by* $\omega$ if for any $n \in {\mathbb {N}}$, $p=1, \ldots , n$ and $i_{1}, \ldots , i_{p} \in {\mathbb {N}}$ with $i_{1} + \cdots + i_{p} = n$, we have

$$\begin{aligned} \begin{aligned}&\Big \vert \text {e}^{ n x_{0} (t) } \int _{ 0 \leqslant u_{1}< \cdots < u_{p} \leqslant t } \text {e}^{-i_{1} x_{0} (u_{1})} \text {d} x_{i_{1}} (u_{1}) \cdots \text {e}^{ - i_{p} x_{0} ( u_{p} ) } \text {d} x_{i_{p}} (u_{p}) \Big \vert \leqslant \frac{ \omega (0,t)^{n} }{ n! }, \end{aligned} \end{aligned}$$

and

$$\begin{aligned} \begin{aligned}&\Big \vert \text {e}^{ n x_{0} (t) } \int _{ 0 \leqslant u_{1}< \cdots< u_{p} \leqslant t } \text {e}^{-i_{1} x_{0} (u_{1})} \text {d} x_{i_{1}} (u_{1}) \cdots \text {e}^{ - i_{p} x_{0} ( u_{p} ) } \text {d} x_{i_{p}} (u_{p}) \\&\qquad - \text {e}^{ n x_{0} (s) } \int _{ 0 \leqslant u_{1}< \cdots < u_{p} \leqslant s } \text {e}^{-i_{1} x_{0} (u_{1})} \text {d} x_{i_{1}} (u_{1}) \cdots \text {e}^{ - i_{p} x_{0} (u_{p}) } \text {d} x_{i_{p}} (u_{p}) \Big \vert \\&\quad \leqslant \omega (s,t) \frac{ \omega (0,T)^{n-1} }{ (n-1)! }, \end{aligned} \end{aligned}$$

for any $$0 \leqslant s \leqslant t \leqslant T$$.

Henceforth, we will refer to Eq. (3.1) as the *Loewner–Kufarev equation controlled by* $\omega$, or the $\omega$-*controlled Loewner–Kufarev equation.*

A natural question to be asked is, how a control function as driving function determines a control function for (3.1). We will give one of the answers in Corollary 4.5.

Let $H = L^{2} ( S^{1},{\mathbb {C}} )$ be the Hilbert space of all square-integrable complex-valued functions on the unit circle $S^{1}$, and we denote by $\text {Gr} := \text {Gr}(H)$ the Segal–Wilson Grassmannian (see [1, Definition 3.1] or [17, Sect. 2]). Any bounded univalent function $f : {\mathbb {D}} \rightarrow {\mathbb {C}}$ with $f(0) = 0$ and $\partial f( {\mathbb {D}} )$ being a Jordan curve, is embedded into $\text {Gr}$ via

$$\begin{aligned} f \mapsto W_{f} := \overline{ \text {span} \big ( \{ 1 \} \cup \{ Q_{n} \circ f \circ (1/z)\vert _{S^{1}} \}_{n \geqslant 1} \big ) }^{H} \in \text {Gr} \end{aligned}$$

(see [1, Sects. 3.2 and 3.3]), where $Q_{n}$ is the *n*-th Faber polynomial associated to *f*.

Note that *f* extends to a continuous function on $\overline{{\mathbb {D}}}$ by Caratheodory’s Extension Theorem for holomorphic functions.

Let $H^{1/2} = H^{1/2}(S^{1})$ be the Sobolev space on $S^{1}$ endowed with the inner product given by $\langle h, g \rangle _{H^{1/2}} = h_{0} {\overline{g}}_{0} + \sum _{n \in {\mathbb {Z}}} \vert n \vert h_{n} {\overline{g}}_{n}$ for $h = \sum _{n \in {\mathbb {Z}}} h_{n} z^{n}, g = \sum _{n \in {\mathbb {Z}}} g_{n} z^{n} \in H^{1/2}$. Assume that *f* extends to a holomorphic function on an open neighbourhood of $\overline{{\mathbb {D}}}$. Then $\text {span} \big ( \{ 1 \} \cup \{ Q_{n} \circ f \circ (1/z)\vert _{S^{1}} \}_{n \geqslant 1} \big ) \subset H^{1/2}$ and we consider the orthogonal projection

$$\begin{aligned} \mathcal {P}_{f}: H^{1/2} \rightarrow W_{f}^{1/2}, \quad \text {where } W_{f}^{1/2} := \overline{ \text {span} \big ( \{ 1 \} \cup \{ Q_{n} \circ f \circ (1/z)\vert _{S^{1}} \}_{n \geqslant 1} \big ) }^{H^{1/2}} \end{aligned}$$

rather than the orthogonal projection $H \rightarrow W_{f}$.

Then, as we prove with Murayama in [2], to every properly bounded control function $\omega$ there exists a unique solution to the Loewner–Kufarev equation which is univalent on the unit disc and can be holomorphically extended across the unit circle. Our main result is

### Theorem 3.3

Suppose that $\omega (0,T) < \frac{1}{8}$. Then there exists a constant $c = c(T) > 0$ such that

$$\begin{aligned} \begin{aligned} \Vert \mathcal {P}_{f_{t}} - \mathcal {P}_{f_{s}} \Vert _{\text {op}} \leqslant c \quad \omega (s,t) \end{aligned} \end{aligned}$$

for every $0 \leqslant s < t \leqslant T$, where $\Vert \bullet \Vert _{\text {op}}$ is the operator norm.

Thus we obtained a continuity result with respect to the time-variable of the solution embedded into the Grassmannian in which the modulus of continuity is measured by the control function $\omega$.

## Auxiliary estimates along controlled Loewner–Kufarev equations

### Controlling Loewner–Kufarev equation by its driving function

We shall begin with a prominent example of a control function as follows.

#### Example 4.1

If $y : [0,+\infty ) \rightarrow {\mathbb {C}}$, is continuous and of bounded variation, then we have

$$\begin{aligned} \begin{aligned} \Vert y \Vert _{1\text {-var}(s,t)} := \sup _{ \begin{array}{c} n \in {\mathbb {N}} ; \\ s = u_{0}< u_{1}< \cdots< u_{n-1}< u_{n}=t \end{array} } \sum _{i=1}^{n} \vert y_{u_{i}} - y_{u_{i-1}} \vert < +\infty \end{aligned} \end{aligned}$$

for every $0 \leqslant s \leqslant t$. Then $\omega (s,t) := \Vert y \Vert _{1-\text {Var}(s,t)}$ defines a control function.

#### Definition 4.2

Let $\omega$ be a control function. We say that a continuous function $y : [0,T] \rightarrow {\mathbb {C}}$ of bounded variation, *is controlled by* $\omega$ if $\Vert y \Vert _{1\text {-var}(s,t)} \leqslant \omega (s,t)$ for every $0 \leqslant s \leqslant t \leqslant T$.

As is well known, introducing control functions makes our calculations stable as follows.

#### Example 4.3

Let $n\in {\mathbb {N}}$ and $y_{1}, \ldots , y_{n} : [0,+\infty ) \rightarrow {\mathbb {C}}$ be continuous and controlled by a control function $\omega$. Then we have

$$\begin{aligned} \begin{aligned}&\sup _{ \begin{array}{c} m \in {\mathbb {N}} ; \\ s = r_{0}< r_{1}< \\ \cdots< r_{m-1}< r_{m}=t \end{array} } \sum _{i=1}^{m} \big \vert \int _{ r_{i-1} \leqslant u_{1}< \cdots < u_{n} \leqslant r_{i} } \text {d} y_{1} (u_{1}) \cdots \text {d} y_{n} (u_{n}) \big \vert \leqslant \frac{ \omega (s,t)^{n} }{ n! } \end{aligned} \end{aligned}$$

for every $$0 \leqslant s \leqslant t$$.

In fact, we shall prove this by induction on *n*. The case for $n=1$ is clear by definition. Consider the case for $n-1$. Putting $\omega _{s}(t) := \omega (s,t)$, we find that the total variation measure $\vert \text {d} y_{n} \vert$ on $[s, +\infty )$ is smaller than the Lebesgue-Stieltjes measure $\text {d} \omega _{s}$ associated with $\omega _{s}$ on $[s, +\infty )$, in the sense of $\int _{B} \vert \text {d} y_{n} \vert \leqslant \int _{B} \text {d} \omega _{s}$ for any Borel set $B \subset [s,+\infty )$. Therefore we have

$$\begin{aligned} \begin{aligned}&\big \vert \int _{ r_{i-1} \leqslant u_{1}< \cdots< u_{n} \leqslant r_{i} } \text {d} y_{1} (u_{1}) \cdots \text {d} y_{n} (u_{n}) \big \vert \\&\quad \leqslant \int _{r_{i-1}}^{r_{i}} \Big \vert \int _{ r_{i-1} \leqslant u_{1}< \cdots < u_{n-1} \leqslant u_{n} } \text {d} y_{1} (u_{1}) \cdots \text {d} y_{n-1} (u_{n-1}) \Big \vert \vert \text {d} y_{n} (u_{n}) \vert \\&\quad \leqslant \int _{r_{i-1}}^{r_{i}} \frac{ \{ \omega _{r_{i-1}} (u) \}^{n-1} }{ (n-1)! } \text {d} \omega _{r_{i-1}} (u) = \frac{ \omega ( r_{i-1}, r_{i} )^{n} }{ n! }. \end{aligned} \end{aligned}$$

Since the control function is nonnegative and super-additive, it holds that

$$\begin{aligned} \sum _{i=1}^{m} \omega ( r_{i-1}, r_{i} )^{n} \leqslant \Big ( \sum _{i=1}^{m} \omega ( r_{i-1}, r_{i} ) \Big )^{n} \leqslant \omega ( s, t ) ^{n}, \end{aligned}$$

and hence we get the above inequality.

#### Proposition 4.4

Let $\omega _{0}$ and $\omega$ be two control functions. Let $x_{0}:[0, +\infty ) \rightarrow {\mathbb {R}}$ be a continuous function controlled by $\omega _{0}$ and with $x_{0} (0) = 0$. Then

(i) $\omega ^{\prime } (s,t) := \text {e}^{\omega _{0}(s,t)} ( \omega _{0} (s,t) + \omega (s,t) ) ,$ for $0 \leqslant s \leqslant t$ defines a control function.

Let $n \in {\mathbb {N}}$ and $y_{1}, \ldots , y_{n} : [ 0, +\infty ) \rightarrow {\mathbb {C}},$ be continuous functions controlled by $\omega$. Then

(ii) we have
  $$\begin{aligned} \begin{aligned} \text {e}^{ n x_{0}(t) } \big \vert \int _{ 0 \leqslant u_{1}< \cdots < u_{n} \leqslant t } \text {d} y_{1} (u_{1}) \cdots \text {d} y_{n} (u_{n}) \big \vert \leqslant \frac{ \omega ^{\prime } (0,t)^{n} }{ n! }. \end{aligned} \end{aligned}$$
(iii) For each $0 \leqslant s \leqslant t \leqslant T$, we have
  $$\begin{aligned} \begin{aligned}&\Big \vert \text {e}^{ n x_{0} (t) } \int _{ 0 \leqslant u_{1}< \cdots< u_{n} \leqslant t } \text {d} y_{1} (u_{1}) \cdots \text {d} y_{n} (u_{n}) \\&\qquad - \text {e}^{ n x_{0} (s) } \int _{ 0 \leqslant u_{1}< \cdots < u_{n} \leqslant s } \text {d} y_{1} (u_{1}) \cdots \text {d} y_{n} (u_{n}) \Big \vert \\&\quad \leqslant \Big ( \omega _{0} (s,t) + \omega (s,t) \Big ) \Big ( \omega ^{\prime } (0,T) + \text {e}^{ \omega _{0} (0,T) } \Big ) \frac{ \omega ^{\prime } (0,T)^{n-1} }{ (n-1)! } . \end{aligned} \end{aligned}$$

#### Proof

(i) It is enough to show the super-additivity. Put $\omega ^{\prime \prime } := \omega _{0} + \omega$. Let $0 \leqslant s \leqslant u \leqslant t$ be arbitrary. Then

$$\begin{aligned} \begin{aligned} \omega ^{\prime } (s,u) + \omega ^{\prime } (u,t)&= \text {e}^{ \omega _{0} (s,u) } \omega ^{ \prime \prime } (s,u) + \text {e}^{ \omega _{0} (u,t) } \omega ^{ \prime \prime } (u,t) \\&= \text {e}^{ \omega _{0} (s,t) } \big \{ \text {e}^{ \omega _{0} (s,u) - \omega _{0} (s,t) } \omega ^{ \prime \prime } (s,u) + \text {e}^{ \omega _{0} (u,t) - \omega _{0} (s,t) } \omega ^{ \prime \prime } (u,t) \big \} . \end{aligned} \end{aligned}$$

By the super-additivity and non-negativity of $\omega _{0}$, we have $\omega _{0} (s,u) - \omega _{0} (s,t) \leqslant 0$ and $\omega _{0} (u,t) - \omega _{0} (s,t) \leqslant 0$. Therefore, by using the non-negativity and super-additivity for $\omega ^{ \prime \prime }$, we get

$$\begin{aligned} \begin{aligned}&\omega ^{\prime } (s,u) + \omega ^{\prime } (u,t) \leqslant \text {e}^{ \omega _{0} (s,t) } \big \{ \omega ^{ \prime \prime } (s,u) + \omega ^{ \prime \prime } (u,t) \big \} \leqslant \text {e}^{ \omega _{0} (s,t) } \omega ^{ \prime \prime } (s,t) = \omega ^{\prime } (s,t). \end{aligned} \end{aligned}$$

(ii) Since $x_{0} = 0$, we have $\text {e}^{n x_{0}(t)} = ( \text {e}^{x_{0}(t) - x_{0}(0)} )^{n} \leqslant \text {e}^{ n \omega _{0} (0,t) }$. On the other hand, by Example 4.3 we have that

$$\begin{aligned} \begin{aligned} \big \vert \int _{ 0 \leqslant u_{1}< \cdots < u_{n} \leqslant t } \text {d} y_{1} (u_{1}) \cdots \text {d} y_{n} (u_{n}) \big \vert \leqslant \frac{ \omega (0,t)^{n} }{ n! }. \end{aligned} \end{aligned}$$

Hence the assertion is immediate.

(iii) Let $0 \leqslant s \leqslant t \leqslant T$ be arbitrary. Then we have

$$\begin{aligned} \begin{aligned}&\Big \vert \text {e}^{ n x_{0} (t) } \int _{ 0 \leqslant u_{1}< \cdots< u_{n} \leqslant t } \text {d} y_{1} (u_{1}) \cdots \text {d} y_{n} (u_{n}) - \text {e}^{ n x_{0} (s) } \int _{ 0 \leqslant u_{1}< \cdots< u_{n} \leqslant s } \text {d} y_{1} (u_{1}) \cdots \text {d} y_{n} (u_{n}) \Big \vert \\&\leqslant \vert \text {e}^{ n x_{0} (s) } - \text {e}^{ n x_{0} (t) } \vert \times \Big \vert \int _{ 0 \leqslant u_{1}< \cdots< u_{n} \leqslant t } \text {d} y_{1} (u_{1}) \cdots \text {d} y_{n} (u_{n}) \Big \vert \\&\quad + \text {e}^{ x_{0} (s) } \int _{s}^{t} \text {e}^{ (n-1) x_{0} (s) } \Big \vert \int _{ 0 \leqslant u_{1}< \cdots < u_{n-1} \leqslant u_{n} } \text {d} y_{1} (u_{1}) \cdots \text {d} y_{n} (u_{n}) \Big \vert \vert \text {d} y_{n} ( u_{n} ) \vert . \end{aligned} \end{aligned}$$

Since it holds that $\vert \text {e}^{ n x_{0} (s) } - \text {e}^{ n x_{0} (t) } \vert \leqslant n \omega _{0} (s,t) \text {e}^{ n \omega _{0} (0,t) },$ and by using (ii), the above quantity is bounded by

$$\begin{aligned} \begin{aligned}&n \omega _{0} (s,t) \text {e}^{ n \omega _{0} (0,t) } \frac{ \omega (0,t)^{n} }{ n! } + \text {e}^{ \omega _{0} (0,t) } \omega (s,t) \frac{ \omega ^{\prime } (0,t)^{n-1} }{ (n-1)! } \\&= \Big ( \omega _{0} (s,t) \omega ^{\prime } (0,t) + \omega (s,t) \text {e}^{ \omega _{0} (0,t) } \Big ) \frac{ \omega ^{\prime } (0,t)^{n-1} }{ (n-1)! } . \end{aligned} \end{aligned}$$

$$\square $$

We shall remark here that the control functions form a convex cone, namely, (a) the sum of two control functions is a control function, (b) any control function multiplied by a positive real constant is again a control function. Therefore, the quantities in Proposition 4.4–(ii, iii) are estimated by using a single control function. Therefore, the following is immediate.

#### Corollary 4.5

Let $\omega _{0}$ and $\omega$ be two control functions. Consider the Loewner–Kufarev equation (3.1) and suppose that the following two conditions hold:

(i) $x_{0}$ is controlled by $\omega _{0}$;
(ii) for every $n \in {\mathbb {N}}$, there exist continuous functions $y_{1}^{n}, y_{2}^{n}, \ldots , y_{n}^{n}: [0,T] \rightarrow {\mathbb {C}}$ controlled by $\omega$ such that
  $$\begin{aligned} x_{n}(t) = \int _{ 0 \leqslant s_{1}< s_{2}< \cdots < s_{n} \leqslant t } \text {d} y_{1}^{n} (s_{1}) \text {d} y_{2}^{n} (s_{2}) \cdots \text {d} y_{n}^{n} (s_{n}), \quad 0 \leqslant t \leqslant T. \end{aligned}$$

Then there exists a constant $c>0$ such that (3.1) is a Loewner–Kufarev equation controlled by $\omega ^{\prime } := c ( \omega _{0} + \omega ) \exp ( \omega _{0} )$.

The following is a consequence of [1, Theorem 2.8] and will be proved in Sect. A.1.

#### Corollary 4.6

Let $\omega$ be a control function and $\{ f_{t} \}_{0 \leqslant t \leqslant T}$ be a solution to the Loewner–Kufarev equation controlled by $\omega$. If $\omega (0,T) < \frac{1}{4}$ then $f_{t} ( {\mathbb {D}} )$ is bounded for any $t \in [0,T]$.

### Some analytic aspects of Grunsky coefficients

Let $S, S^{\prime } \subset {\mathbb {Z}}$ be countably infinite subsets, and $A=(a_{i,j})_{i \in S, j \in S^{\prime }}$ be an $S \times S^{\prime }$-matrix. For each sequence $x = (x_{j})_{j \in S^{\prime }}$ of complex numbers, we define a sequence $T_{A} x = ( (T_{A}x)_{i} )_{i \in S}$ by $(T_{A}x)_{i} := \sum _{j \in S^{\prime }} a_{ij} x_{j}$ if it converges for all $i \in S$. We will still denote $T_{A}x$ by *Ax* when it is defined.

Let $\ell _{2} (S)$ be the Hilbert space consisting of all sequences $a = (a_{i})_{i \in S}$ such that $\sum _{i \in S} \vert a_{i} \vert ^{2} < +\infty$, with the Hermitian inner product $\langle a, b \rangle _{2} = \sum _{i \in S} a_{i} \overline{b_{i}},$ for $a=(a_{i})_{i \in S}$, $b=(b_{i})_{i \in S} \in \ell _{2} (S)$. The associated norm will be denoted by $\Vert \bullet \Vert _{2}$.

For each $s \in {\mathbb {R}}$, the space

$$\begin{aligned} \ell _{2}^{ s } (S) := \Big \{ a = (a_{n})_{n \in S} : \sum _{n \in S} (1 + n^{2} )^{ s } \vert a_{n} \vert ^{2} < +\infty \Big \} \end{aligned}$$

is a Hilbert space under the Hermitian inner product given by

$$\begin{aligned} \langle a, b \rangle _{2,s} := \sum _{n \in S} \max \{ 1, \vert n \vert \}^{2s} a_{n} \overline{b_{n}} \end{aligned}$$

for $a=(a_{i})_{i \in S}$, $b=(b_{i})_{i \in S} \in \ell _{2}^{ s } (S)$. The associated norm will be denoted by $\Vert \bullet \Vert _{2, s }$.

Let us recall a classical and well-known result from the theory of univalent functions. For the definition and properties of Grunsky coefficients, see [20, Chapter 2, Sect. 2], [21, Sect. 2.2] or [1, Definition A.1 and Proposition A.2].

#### Theorem 4.7

(Grunsky’s inequality [16, Theorem 3.2]) Let $f : {\mathbb {D}} \rightarrow {\mathbb {C}}$ be a univalent functions with $f(0) = 0$, and let $(b_{m,n})_{m,n \leqslant -1}$ be the Grunsky coefficients associated to *f*. Then for any $m \in {\mathbb {N}}$ and $\lambda _{-m}, \lambda _{-m+1} , \ldots , \lambda _{-1} \in {\mathbb {C}}$, it holds that

$$\begin{aligned} \begin{aligned} \sum _{k \leqslant -1} (-k) \Big \vert \sum _{l=-m}^{-1} b_{k,l} \lambda _{l} \Big \vert ^{2} \leqslant \sum _{k=-m}^{-1} \frac{ \vert \lambda _{k} \vert ^{2} }{ (-k) } . \end{aligned} \end{aligned}$$

This can be rephrased with our notation as follows: Let $B := ( \sqrt{m(-n)} b_{-m,n})_{m \in {\mathbb {N}}, n \in -{\mathbb {N}}}$, where $b_{m,n}$ for $m,n \leqslant -1$ are Grunsky coefficients associated to a univalent function *f* on ${\mathbb {D}}$ such that $f(0) = 0$.

#### Corollary 4.8

(i) $B : \ell _{2} (-{\mathbb {N}}) \rightarrow \ell _{2} ({\mathbb {N}})$ and is a bounded linear operator with the operator norm satisfying $\Vert B \Vert \leqslant 1$.
(ii) $B^{*} : \ell _{2} ({\mathbb {N}}) \rightarrow \ell _{2} (-{\mathbb {N}})$ and is a bounded linear operator with the operator norm satisfying $\Vert B^{*} \Vert \leqslant 1$.
(iii) The bounded linear operator $1 + B_{t} B_{t}^{*} : \ell _{2} ({\mathbb {N}}) \rightarrow \ell _{2} ({\mathbb {N}})$ is injective and has a dense image.

#### Proof

(i) For each $a = ( \ldots , a_{-3}, a_{-2}, a_{-1} ) \in \ell _{2} ( -{\mathbb {N}} )$, we have by Theorem 4.7,

$$\begin{aligned} \begin{aligned} \Vert B a \Vert _{2}^{2}&= \sum _{n=1}^{\infty } \Big ( \sum _{k=1}^{\infty } \sqrt{nk}\, b_{-n,-k} a_{-k} \Big ) \Big ( \overline{ \sum _{l=1}^{\infty } \sqrt{nl}\, b_{-n,-l} a_{-l} } \Big ) \\&= \sum _{n=1}^{\infty } n \Big \vert \sum _{k=1}^{\infty } b_{-n,-k} ( \sqrt{k}\, a_{-k} ) \Big \vert ^{2} \leqslant \sum _{n=1}^{\infty } \frac{ \vert \sqrt{n}\, a_{-n} \vert ^{2} }{ n } = \Vert a \Vert _{2}^{2}. \end{aligned} \end{aligned}$$

(ii) Since the Grunsky matrix $(b_{m,n})_{m,n \leqslant -1}$ is symmetric: $b_{m,n} = b_{n,m}$ for all $m,n \leqslant -1$, the assertion is proved similarly to (i).

(iii) The injectivity is clear since the adjoint operator of *B* is $B^{*}$. Then the second assertion is also clear since $1+BB^{*}$ is self-adjoint. $\square$

#### Remark 4.9

The semi-infinite matrix defined by $B_{1} := ( \sqrt{mn} b_{-m,-n} )_{m,n \in {\mathbb {N}}}$ is called the *Grunsky operator*, and then the Grunsky inequality (Theorem 4.7) shows that $B_{1}$ is a bounded operator on $\ell _{2} ({\mathbb {N}})$ with operator norm $\leqslant 1$. This operator, together with three additional Grunsky operators, are known to play a fundamental role in the study of the geometry of the universal Teichmüller space. For details, cf. the papers by Takhtajan-Teo [20] or Krushkal [11].

In the sequel, we fix a control function $\omega$, and a solution $\{ f_{t} \}_{0 \leqslant t \leqslant T}$ to a Loewner–Kufarev equation controlled by $\omega$. We denote by $b_{m,n}(t)$ for $m,n \leqslant -1$ the Grunsky coefficients associated with $f_{t}$, and

$$\begin{aligned} \begin{aligned} B_{t}&:= \big ( \sqrt{m (-n)}\, b_{-m,n}(t) \big )_{m \in {\mathbb {N}}, n \in -{\mathbb {N}}}, \\ B_{t}^{*}&:= \big ( \sqrt{(-n) m}\, b_{ n, -m }(t) \big )_{ n \in -{\mathbb {N}}, m \in {\mathbb {N}} }. \end{aligned} \end{aligned}$$

It is clear that the linear operator $( 1 + B_{t} B_{t}^{*} )^{-1} : \text {Im}( 1 + B_{t} B_{t}^{*} ) \rightarrow \ell _{2} ({\mathbb {N}})$ is bounded. Therefore by Corollary 4.8–(iii), $( 1 + B_{t} B_{t}^{*} )^{-1}$ extends to $\ell _{2} ({\mathbb {N}})$ and the extension will be denoted by $A_{t} : \ell _{2} ({\mathbb {N}}) \rightarrow \ell _{2} ({\mathbb {N}})$. In particular, it is easy to see that $\Vert A_{t} \Vert \leqslant 1,$ holds for the operator norm.

We shall exhibit the indices which parametrise our operators in order to help understanding the following:

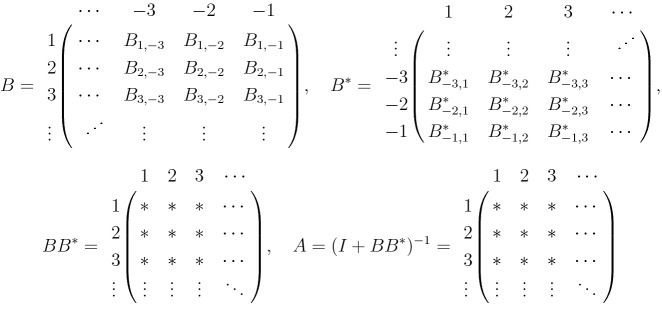



and

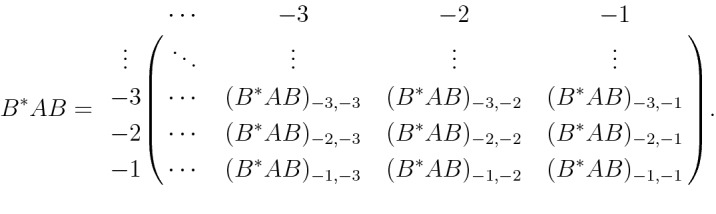



The following is a consequence from [1, Theorem 2.12] and will be proved in Sect. A.2.

#### Corollary 4.10

Let $$\omega $$ be a control function, and $$\{ f_{t} \}_{0 \leqslant t \leqslant T}$$ be a solution to the Loewner–Kufarev equation controlled by $$\omega $$. Let $$ b_{-m,-n}(t)$$, $$n,m \in {\mathbb {N}} $$ be the Grunsky coefficients associated to $$f_{t}$$, for $$0 \leqslant t \leqslant T$$. Then for any $$0 \leqslant s \leqslant t \leqslant T$$ and $$n,m \in {\mathbb {N}}$$ with $$n+m \geqslant 3$$, we have

(i) $$ \vert b_{-1,-1} (t) \vert \leqslant \frac{ \omega (0,t)^{2} }{ 2 } $$ and $$ \vert b_{-1,-1} (t) - b_{-1,-1} (s) \vert \leqslant \omega (s,t) \omega (0,T) $$.
(ii) $$\displaystyle \vert b_{-m,-n} (t) \vert \leqslant \frac{ ( 8 \omega (0,t) )^{m+n} }{ 16 (m+n) (m+n-1) (m+n-2) } $$.
(iii) $$\displaystyle \vert b_{-m,-n} (t) - b_{-m,-n} (s) \vert \leqslant \frac{ \omega (s,t) ( 8 \omega (0,T) )^{m+n-1} }{ 16 (m+n-1) (m+n-2) } $$.

Along the Loewner–Kufarev equation controlled by $\omega$, we obtain the following

#### Corollary 4.11

If $\omega (0,T) < \frac{1}{8}$, then for $0 \leqslant s < t \leqslant T$,

(i) $$\displaystyle \Vert B_{t}^{*} - B_{s}^{*} \Vert = \Vert B_{t} - B_{s} \Vert \leqslant c \omega (s,t) $$,
(ii) $$\displaystyle \Vert A_{t} - A_{s} \Vert \leqslant 2c  \omega (s,t) $$,

where $$ c := \frac{ 8 \omega (0,T) }{ 1 - ( 8 \omega (0,T) )^{2} } > 0 $$.

#### Proof

(i) By Corollary 4.10–(ii), we have

$$\begin{aligned} \begin{aligned}&\Vert B_{t} - B_{s} \Vert ^{2} \leqslant \sum _{n=1}^{\infty } \sum _{m=1}^{\infty } \vert ( B_{t} - B_{s} )_{n,-m} \vert ^{2} \\&= \sum _{n=1}^{\infty } \sum _{m=1}^{\infty } \vert \sqrt{nm} ( b_{-n.-m} (t) - b_{-n,-m} (s) ) \vert ^{2} \\&\leqslant \sum _{n=1}^{\infty } \sum _{m=1}^{\infty } \Big \vert \frac{ \sqrt{nm} \omega (s,t) ( 8 \omega (0,T) )^{n+m-1} }{ 16 (n+m-1) (n+m-2) } \Big \vert ^{2} \\&\leqslant \Big ( \frac{ \omega (s,t) }{ 8 \omega (0,T) } \Big )^{2} \Big ( \sum _{n=1}^{\infty } ( 8 \omega (0,T) )^{2n} \Big )^{2} = \Big ( \omega (s,t) \frac{ 8 \omega (0,T) }{ 1 - ( 8 \omega (0,T) )^{2} } \Big )^{2} . \end{aligned} \end{aligned}$$

(ii) Since

$$\begin{aligned} \begin{aligned}&A_{t} - A_{s} = ( 1 + B_{t} B_{t}^{*} )^{-1} - ( 1 + B_{s} B_{s}^{*} )^{-1} \\&\quad = ( 1 + B_{t} B_{t}^{*} )^{-1} ( B_{s} B_{s}^{*} - B_{t} B_{t}^{*} ) ( 1 + B_{s} B_{s}^{*} )^{-1} \\&\quad = ( 1 + B_{t} B_{t}^{*} )^{-1} ( B_{s} - B_{t} ) B_{t}^{*} ( 1 + B_{s} B_{s}^{*} )^{-1} \\&\qquad - ( 1 + B_{t} B_{t}^{*} )^{-1} B_{s} ( B_{t}^{*} - B_{s}^{*} ) ( 1 + B_{s} B_{s}^{*} )^{-1}, \end{aligned} \end{aligned}$$

we have $$ \Vert A_{t} - A_{s} \Vert \leqslant \Vert B_{s} - B_{t} \Vert + \Vert B_{s}^{*} - B_{t}^{*} \Vert = 2 \Vert B_{t} - B_{s} \Vert $$. $$\square $$

Finally, define $\Lambda = ( \Lambda _{m,n} )_{ m \in {\mathbb {Z}}, n \in {\mathbb {Z}} }$ by $\Lambda _{m,n} := \sqrt{m}\, \delta _{m,-n} + \delta _{m,0} \delta _{0,n}$ for $m \in {\mathbb {N}}$ and $n \in -{\mathbb {N}}$, that is,4.1It is clear that $\Lambda : \ell _{2}^{ 1/2 } ( {\mathbb {Z}} ) \rightarrow \ell _{2} ( {\mathbb {Z}} )$ and is a continuous linear isomorphism.

## Proof of Theorem 3.3

Let $\omega$ be a control function such that $\omega (0,T) < \frac{1}{8}$, and let $\{ f_{t} \}_{0 \leqslant t \leqslant T}$ be a univalent solution to the Loewner–Kufarev equation controlled by $\omega$.

Recall, that by the results in [2], $f_{t}$ extends to a holomorphic function on an open neighbourhood of $\overline{{\mathbb {D}}}$ for all $t \in [0,T]$.

We then note that for each $t \in [0,T]$, it holds that $Q_{n} ( t, f_{t} (1/z) )\vert _{S^{1}} \in H^{1/2}$, where $Q_{n} ( t, w )$ is the *n*-th Faber polynomial associated to $f_{t}$. Therefore we have

$$\begin{aligned} \text {span} \big ( \{ 1 \} \cup \{ Q_{n} ( t, f_{t} (1/z) )\vert _{S^{1}} \}_{n \geqslant 1} \big ) \subset H^{1/2} \subset H. \end{aligned}$$

In particular, we have

$$\begin{aligned} \begin{aligned} W_{f_{t}}^{1/2}&:= \overline{ \text {span} \big ( \{ 1 \} \cup \{ Q_{n} ( t, f_{t} (1/z) )\vert _{S^{1}} \}_{n \geqslant 1} \big ) }^{H^{1/2}} \\&\subset \overline{ \text {span} \big ( \{ 1 \} \cup \{ Q_{n} ( t, f_{t} (1/z) )\vert _{S^{1}} \}_{n \geqslant 1} \big ) }^{H} = W_{f_{t}}. \end{aligned} \end{aligned}$$

We fix an inner product on $H^{1/2}$ by requiring for $h = \sum _{n \in {\mathbb {Z}}} h_{n} z^{n}, g = \sum _{n \in {\mathbb {Z}}} g_{n} z^{n} \in H^{1/2}$, that $\langle h, g \rangle _{H^{1/2}} := h_{0} {\overline{g}}_{0} + \sum _{n=1}^{\infty } n ( h_{-n} {\overline{g}}_{-n} + h_{n} {\overline{g}}_{n} )$. Then $\{ \frac{ z^{-n} }{ \sqrt{n} } \}_{n \in {\mathbb {N}}} \cup \{ 1 \} \cup \{ \frac{ z^{n} }{ \sqrt{n} } \}_{n \in {\mathbb {N}}}$ forms a complete orthonormal system of $H^{1/2}$. By this, the infinite matrix $\Lambda$ defined in (4.1) determines a bounded linear isomorphism $H^{1/2} \rightarrow H$ through the identification $H^{1/2} \cong \ell _{2}^{1/2}({\mathbb {Z}})$.

Recall that for each univalent function $f : {\mathbb {D}} \rightarrow {\mathbb {C}}$ with $f(0) = 0$ and an analytic continuation across $S^{1}$, the orthogonal projection $H^{1/2} \rightarrow W_{f}^{1/2}$ is denoted by $\mathcal {P}_{f}$. In order to prove Theorem 3.3, we need to calculate the projection operator $\mathcal {P}_{f}$. For this, we shall consider first the following change of basis.

Let $\mathbf{w }_{n} (z) := Q_{n} \circ f (z^{-1})$, for $z \in S^{1}$ and $n \in {\mathbb {N}}$. Then we have5.1By putting

$$\begin{aligned} \begin{aligned} \widetilde{\mathbf{z }}_{+}&:= \Big ( \ldots , \frac{z^{3}}{\sqrt{3}}, \frac{z^{2}}{\sqrt{2}}, \frac{z}{\sqrt{1}} \Big ), \quad \widetilde{\mathbf{z }}_{-} := \Big ( \frac{z^{-1}}{\sqrt{1}}, \frac{z^{-2}}{\sqrt{2}}, \frac{ z^{-3} }{ \sqrt{3} }, \ldots \Big ), \end{aligned} \end{aligned}$$

and

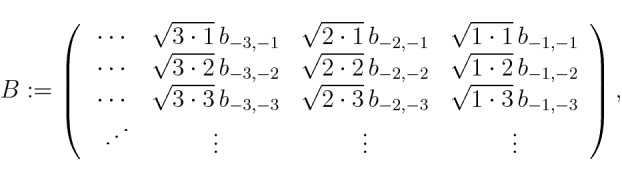



(so we have put $B_{t} = ( (B_{t})_{n,m} )_{n \geqslant 1, m \leqslant -1}$ where $(B_{t})_{n,m} := \sqrt{n(-m)}\, b_{m,-n} (t)$ for $n \geqslant 1$ and $m \leqslant -1$), the Eq. (5.1) is written in a simpler form as:

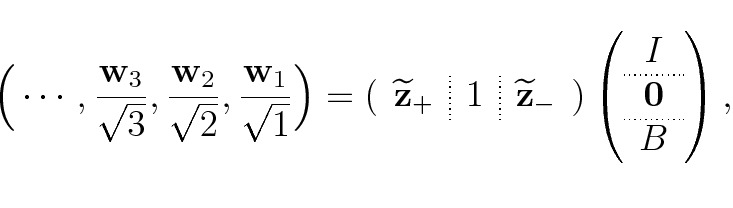



where

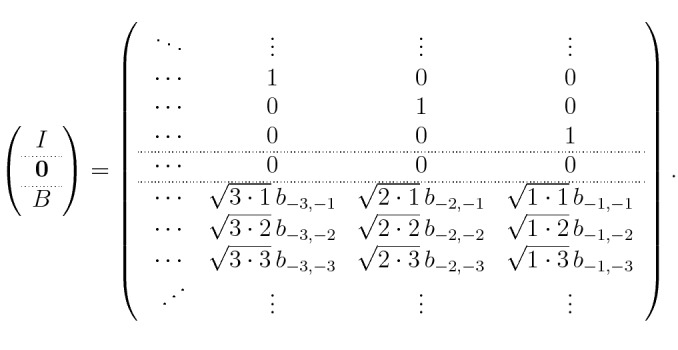



Consider the change of basis

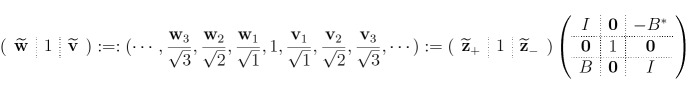



where we note that the matrix on the right-hand side is non-degenerate, with inverse

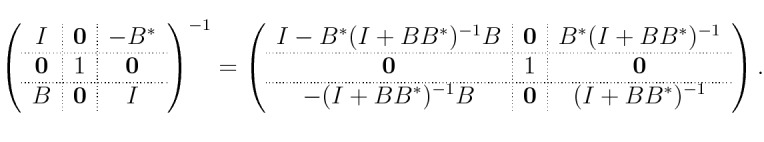



We note that the identity

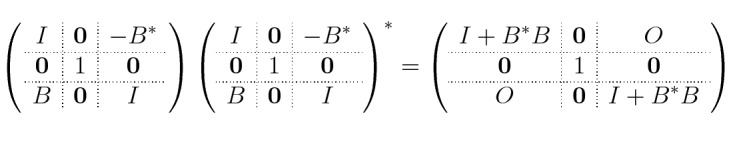



and the fact that $( \widetilde{\mathbf{z }}_{+}, 1, \widetilde{\mathbf{z }}_{-} )$ is a complete orthonormal system in $H^{1/2}$ implies that

$$\begin{aligned} \begin{aligned}&H^{1/2} = \overline{ \text {span} \{ 1, \mathbf{w }_{1}, \mathbf{w }_{2}, \mathbf{w }_{3}, \ldots \} }^{H^{1/2}} \oplus \overline{ \text {span} \{ \mathbf{v }_{1}, \mathbf{v }_{2}, \mathbf{v }_{3}, \ldots \} }^{H^{1/2}} \end{aligned} \end{aligned}$$

is an orthogonal decomposition of $H^{1/2}$.

Let $$A := ( I + BB^{*} )^{-1}$$. Then

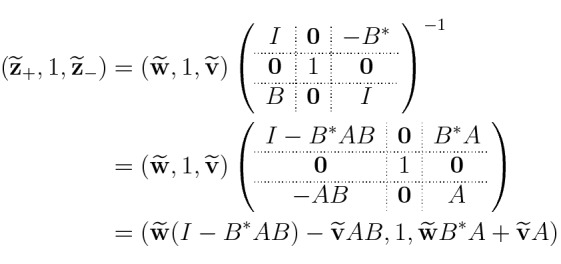



so that

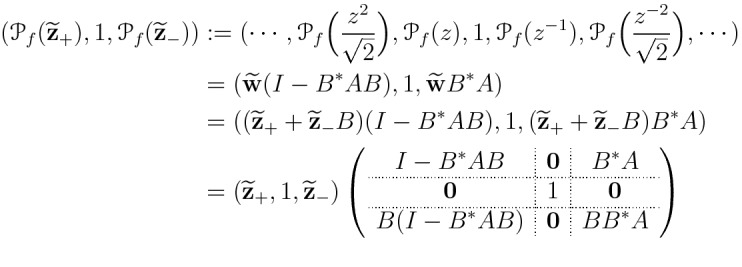



From these, we find that for $n \geqslant 1$,

$$\begin{aligned} \begin{aligned}&\mathcal {P}_{f} \Big ( \frac{ z^{n} }{ \sqrt{n} } \Big ) = \sum _{k=1}^{\infty } \Big \{ \frac{ z^{k} }{ \sqrt{k} } ( I - B^{*} A B )_{-k,-n} + \frac{ z^{-k} }{ \sqrt{k} } ( B ( I - B^{*} A B ) )_{k,-n} \Big \} , \\&\mathcal {P}_{f} \Big ( \frac{ z^{-n} }{ \sqrt{n} } \Big ) = \sum _{k=1}^{\infty } \Big \{ \frac{ z^{k} }{ \sqrt{k} } ( B^{*} A )_{-k,n} + \frac{ z^{-k} }{ \sqrt{k} } ( B B^{*} A )_{k,n} \Big \} , \end{aligned} \end{aligned}$$

from which, the following is immediate:

### Proposition 5.1

Let $$ h = \sum _{k \in {\mathbb {Z}}} h_{k} z^{k} \in H^{1/2} $$. Then

$$\begin{aligned} \begin{aligned} \mathcal {P}_{f} (h) =&\sum _{n=1}^{\infty } \Big \{ \sum _{k=1}^{\infty } \big [ ( I - B^{*} A B )_{-n,-k} \sqrt{k}\, h_{k} + ( B^{*} A )_{-n,k} \sqrt{k}\, h_{-k} \big ] \Big \} \frac{ z^{n} }{ \sqrt{n} } \\&+ h_{0} + \sum _{n=1}^{\infty } \Big \{ \sum _{k=1}^{\infty } \big [ ( B ( I - B^{*} A B ) )_{n,-k} \sqrt{k}\, h_{k} + ( B B^{*} A )_{n,k} \sqrt{k}\, h_{-k} \big ] \Big \} \frac{ z^{-n} }{ \sqrt{n} }. \end{aligned} \end{aligned}$$

Denote by $B_{t}$ the associated matrix of Grunsky coefficients of $f_{t}$.

### Proposition 5.2

Let $$ h = \sum _{k \in {\mathbb {Z}}} h_{k} z^{k} \in H^{1/2} $$. Then

$$\begin{aligned} \begin{aligned}&\mathcal {P}_{f_{t}} (h) - \mathcal {P}_{f_{s}} (h) \\&\quad = \sum _{n=1}^{\infty } \Big \{ \sum _{k=1}^{\infty } ( B_{s}^{*} A_{s} B_{s} - B_{t}^{*} A_{t} B_{t} )_{-n,-k} \sqrt{k}\, h_{k} + \sum _{k=1}^{\infty } ( B_{t}^{*} A_{t} - B_{s}^{*} A_{s} )_{-n,k} \sqrt{k}\, h_{-k} \Big \} \frac{ z^{n} }{ \sqrt{n} } \\&\qquad + \sum _{n=1}^{\infty } \Big \{ \sum _{k=1}^{\infty } \big ( B_{t} ( I - B_{t}^{*} A_{t} B_{t} ) - B_{s} ( I - B_{s}^{*} A_{s} B_{s} ) \big )_{n,-k} \sqrt{k}\, h_{k} \\&\qquad + \sum _{k=1}^{\infty } ( B_{t} B_{t}^{*} A_{t} - B_{s} B_{s}^{*} A_{s} )_{n,k} \sqrt{k}\, h_{-k} \Big \} \frac{ z^{-n} }{ \sqrt{n} } , \end{aligned} \end{aligned}$$

so that

$$\begin{aligned} \begin{aligned}&\Vert \mathcal {P}_{f_{t}} (h) - \mathcal {P}_{f_{s}} (h) \Vert _{ H^{1/2} }^{2} \\&\quad = \sum _{n=1}^{\infty } \Big \vert \sum _{k=1}^{\infty } ( B_{s}^{*} A_{s} B_{s} - B_{t}^{*} A_{t} B_{t} )_{-n,-k} \sqrt{k}\, h_{k} + \sum _{k=1}^{\infty } ( B_{t}^{*} A_{t} - B_{s}^{*} A_{s} )_{-n,k} \sqrt{k}\, h_{-k} \Big \vert ^{2} \\&\qquad + \sum _{n=1}^{\infty } \Big \vert \sum _{k=1}^{\infty } \big ( B_{t} ( I - B_{t}^{*} A_{t} B_{t} ) - B_{s} ( I - B_{s}^{*} A_{s} B_{s} ) \big )_{n,-k} \sqrt{k}\, h_{k} \\&\qquad + \sum _{k=1}^{\infty } ( B_{t} B_{t}^{*} A_{t} - B_{s} B_{s}^{*} A_{s} )_{n,k} \sqrt{k}\, h_{-k} \Big \vert ^{2} . \end{aligned} \end{aligned}$$

We are now in a position to prove Theorem 3.3.

### Proof of Theorem 3.3

By Proposition 5.2, we have

$$\begin{aligned} \begin{aligned}&\Vert \mathcal {P}_{f_{t}} (h) - \mathcal {P}_{f_{s}} (h) \Vert _{ H^{1/2} }^{2} \leqslant 2 ( I + I\!\!I ) + 3 ( I\!\!I\!\!I + I\!V + V ) , \end{aligned} \end{aligned}$$

where

$$\begin{aligned} \begin{aligned} I&:= \sum _{n=1}^{\infty } \Big \vert \sum _{k=1}^{\infty } ( B_{s}^{*} A_{s} B_{s} - B_{t}^{*} A_{t} B_{t} )_{-n,-k} \sqrt{k}\, h_{k} \Big \vert ^{2}, \\ I\!\!I&:= \sum _{n=1}^{\infty } \Big \vert \sum _{k=1}^{\infty } ( B_{t}^{*} A_{t} - B_{s}^{*} A_{s} )_{-n,k} \sqrt{k}\, h_{-k} \Big \vert ^{2}, \\ I\!\!I\!\!I&:= \sum _{n=1}^{\infty } \Big \vert \sum _{k=1}^{\infty } ( B_{t} - B_{s} )_{n,-k} \sqrt{k}\, h_{k} \Big \vert ^{2}, \\ I\!V&:= \sum _{n=1}^{\infty } \Big \vert \sum _{k=1}^{\infty } ( B_{t}B_{t}^{*} A_{t} B_{t} - B_{s}B_{s}^{*} A_{s} B_{s} )_{n,-k} \sqrt{k}\, h_{k} \Big \vert ^{2}, \\ V&:= \sum _{n=1}^{\infty } \Big \vert \sum _{k=1}^{\infty } ( B_{t} B_{t}^{*} A_{t} - B_{s} B_{s}^{*} A_{s} )_{n,k} \sqrt{k}\, h_{-k} \Big \vert ^{2} . \end{aligned} \end{aligned}$$

$$\underline{{ Estimate\, of\, I.}}$$

$$\begin{aligned} \begin{aligned} I&= \sum _{n=1}^{\infty } \Big \vert \sum _{k=1}^{\infty } ( B_{s}^{*} A_{s} B_{s} - B_{t}^{*} A_{t} B_{t} )_{-n,-k} \sqrt{k}\, h_{k} \Big \vert ^{2} \end{aligned} \end{aligned}$$

We shall note that $A_{s} - A_{t} = A_{t} [ ( I + B_{t}B_{t}^{*} ) - ( I + B_{s}B_{s}^{*} ) ] A_{s} = A_{t} ( B_{t}B_{t}^{*} - B_{s}B_{s}^{*} ) A_{s}$ and hence we obtain the following identity:

$$\begin{aligned} \begin{aligned}&B_{s}^{*} A_{s} B_{s} - B_{t}^{*} A_{t} B_{t} \\&\quad = ( B_{s}^{*} - B_{t}^{*} ) A_{s} B_{s} + B_{t}^{*} ( A_{s} - A_{t} ) B_{s} + B_{t}^{*} A_{t} ( B_{s} - B_{t} ) . \end{aligned} \end{aligned}$$

According to this decomposition, *I* can be estimated as

$$\begin{aligned} I \leqslant 3 ( I_{1} + I_{2} + I_{3} ), \end{aligned}$$

where

$$\begin{aligned} \begin{aligned} I_{1}&:= \sum _{n=1}^{\infty } \Big \vert \sum _{k=1}^{\infty } ( ( B_{s}^{*} - B_{t}^{*} ) A_{s} B_{s} )_{-n,-k} \sqrt{k}\, h_{k} \Big \vert ^{2}, \\ I_{2}&:= \sum _{n=1}^{\infty } \Big \vert \sum _{k=1}^{\infty } ( B_{t}^{*} ( A_{s} - A_{t} ) B_{s} )_{-n,-k} \sqrt{k}\, h_{k} \Big \vert ^{2}, \\ I_{3}&:= \sum _{n=1}^{\infty } \Big \vert \sum _{k=1}^{\infty } ( B_{t}^{*} A_{t} ( B_{s} - B_{t} ) )_{-n,-k} \sqrt{k}\, h_{k} \Big \vert ^{2} . \end{aligned} \end{aligned}$$

Each of which is estimated as follows: By Corollaries 4.8 and 4.11, we have

$$\begin{aligned} \begin{aligned} I_{1}&\leqslant \Vert B_{s} - B_{t} \Vert ^{2} \Vert A_{s} B_{s} \Vert ^{2} \Vert \Lambda h \Vert _{H}^{2} \leqslant c_{11} \omega (s,t)^{2} \Vert h \Vert _{ H^{1/2} }^{2}. \end{aligned} \end{aligned}$$

for some constant $c_{11} >0$. Similarly, we have

$$\begin{aligned} \begin{aligned} I_{2}&\leqslant \Vert B_{t}^{*} \Vert ^{2} \Vert A_{s} - A_{t} \Vert ^{2} \Vert B_{t} \Vert ^{2} \Vert \Lambda h \Vert _{H}^{2} \leqslant c_{12} \omega (s,t)^{2} \Vert h \Vert _{ H^{1/2} }^{2}, \\ I_{3}&\leqslant \Vert B_{t} A_{t} \Vert ^{2} \Vert B_{s} - B_{t} \Vert ^{2} \Vert \Lambda h \Vert _{H}^{2} \leqslant c_{13} \omega (s,t)^{2} \Vert h \Vert _{ H^{1/2} }^{2} \end{aligned} \end{aligned}$$

for some constants $c_{12}, c_{13} > 0$. Combining these together, we obtain

$$\begin{aligned} I_{1} \leqslant c_{1} \omega (s,t)^{2} \Vert h \Vert _{ H^{1/2} }^{2} \end{aligned}$$

for some constant $c_{1} > 0$.

$$\underline{{ Estimate\, of\, I\!\!I.}}$$

$$\begin{aligned} \begin{aligned} I\!\!I&= \sum _{n=1}^{\infty } \Big \vert \sum _{k=1}^{\infty } ( B_{t}^{*} A_{t} - B_{s}^{*} A_{s} )_{-n,k} \sqrt{k}\, h_{-k} \Big \vert ^{2} \end{aligned} \end{aligned}$$

According to the identity

$$\begin{aligned} \begin{aligned} B_{t}^{*} A_{t} - B_{s}^{*} A_{s} = ( B_{t}^{*} - B_{s}^{*} ) A_{t} + B_{s}^{*} ( A_{t} - A_{s} ) , \end{aligned} \end{aligned}$$

we estimate $I\!\!I$ as

$$\begin{aligned} \begin{aligned} I\!\!I \leqslant 2( I\!\!I_{1} + I\!\!I_{2} ), \end{aligned} \end{aligned}$$

where

$$\begin{aligned} \begin{aligned} I\!\!I_{1}&= \sum _{n=1}^{\infty } \Big \vert \sum _{k=1}^{\infty } ( ( B_{t}^{*} - B_{s}^{*} )A_{t} )_{-n,k} \sqrt{k}\, h_{-k} \Big \vert ^{2}, \\ I\!\!I_{2}&= \sum _{n=1}^{\infty } \Big \vert \sum _{k=1}^{\infty } ( B_{s}^{*} ( A_{t} - A_{s} ) )_{-n,k} \sqrt{k}\, h_{-k} \Big \vert ^{2} . \end{aligned} \end{aligned}$$

By Corollaries 4.8 and 4.11, we have

$$\begin{aligned} \begin{aligned} I\!\!I_{1}&\leqslant \Vert B_{t}^{*} - B_{s}^{*} \Vert ^{2} \Vert A_{t} \Vert ^{2} \Vert \Lambda h \Vert _{H}^{2} \leqslant c_{21} \omega (s,t)^{ 2 } \Vert h \Vert _{ H^{1/2} }^{2}, \\ I\!\!I_{2}&\leqslant \Vert B_{s}^{*} \Vert ^{2} \Vert A_{t} - A_{s} \Vert ^{2} \Vert \Lambda h \Vert _{H}^{2} \leqslant c_{22} \omega (s,t)^{ 2 } \Vert h \Vert _{ H^{1/2} }^{2}, \end{aligned} \end{aligned}$$

for some constant $c_{21}, c_{22} > 0$. Therefore we have obtained

$$\begin{aligned} I\!\!I \leqslant c_{2} \omega (s,t)^{ 2 } \Vert h \Vert _{ H^{1/2} }^{2} \end{aligned}$$

for some $$c_{2} > 0$$.

$$\underline{{ Estimate\, of\, I\!\!I\!\!I.}}$$

$$\begin{aligned} \begin{aligned} I\!\!I\!\!I&= \sum _{n=1}^{\infty } \Big \vert \sum _{k=1}^{\infty } ( B_{t} - B_{s} )_{n,-k} \sqrt{k}\, h_{k} \Big \vert ^{2} \end{aligned} \end{aligned}$$

is estimated by using Corollary 4.11 as

$$\begin{aligned} \begin{aligned} I\!\!I\!\!I&\leqslant \Vert B_{t} - B_{s} \Vert ^{2} \Vert \Lambda h \Vert _{H}^{2} \leqslant c_{3} \omega (s,t)^{2} \Vert h \Vert _{ H^{1/2} }^{2} \end{aligned} \end{aligned}$$

for some constant $c_{3} > 0$.

$$\underline{{ Estimate\, of\, I\!V.}}$$

$$\begin{aligned} \begin{aligned} I\!V&= \sum _{n=1}^{\infty } \Big \vert \sum _{k=1}^{\infty } ( B_{t}B_{t}^{*} A_{t} B_{t} - B_{s}B_{s}^{*} A_{s} B_{s} )_{n,-k} \sqrt{k}\, h_{k} \Big \vert ^{2} \end{aligned} \end{aligned}$$

Along the decomposition

$$\begin{aligned} \begin{aligned}&B_{t}B_{t}^{*} A_{t} B_{t} - B_{s}B_{s}^{*} A_{s} B_{s} \\&\quad = ( B_{t} - B_{s} ) B_{t}^{*} A_{t} B_{t} + B_{s} ( B_{t}^{*} A_{t} B_{t} - B_{s}^{*} A_{s} B_{s} ) \\&\quad = ( B_{t} - B_{s} ) B_{t}^{*} A_{t} B_{t} + B_{s} ( B_{t}^{*} - B_{s}^{*} ) A_{t} B_{t} + B_{s} B_{s}^{*} A_{s} B_{s} ( B_{s}^{*} - B_{t}^{*} ) A_{t} B_{t} \\&\qquad + B_{s} B_{s}^{*} A_{s} ( B_{s} - B_{t} ) B_{t}^{*} A_{t} B_{t} + B_{s} B_{s}^{*} A_{s} ( B_{t} - B_{s} ) , \end{aligned} \end{aligned}$$

the quantity $I\!V$ is estimated as

$$\begin{aligned} I\!V \leqslant 5 ( I\!V_{1} + I\!V_{2} + I\!V_{3} + I\!V_{4} + I\!V_{5} ), \end{aligned}$$

where

$$\begin{aligned} \begin{aligned} I\!V_{1}&= \sum _{n=1}^{\infty } \Big \vert \sum _{k=1}^{\infty } ( ( B_{t} - B_{s} ) B_{t}^{*} A_{t} B_{t} )_{n,-k} \sqrt{k}\, h_{k} \Big \vert ^{2} , \\ I\!V_{2}&= \sum _{n=1}^{\infty } \Big \vert \sum _{k=1}^{\infty } ( B_{s} ( B_{t}^{*} - B_{s}^{*} ) A_{t} B_{t} )_{n,-k} \sqrt{k}\, h_{k} \Big \vert ^{2} , \\ I\!V_{3}&= \sum _{n=1}^{\infty } \Big \vert \sum _{k=1}^{\infty } ( B_{s} B_{s}^{*} A_{s} B_{s} ( B_{s}^{*} - B_{t}^{*} ) A_{t} B_{t} )_{n,-k} \sqrt{k}\, h_{k} \Big \vert ^{2} , \\ I\!V_{4}&= \sum _{n=1}^{\infty } \Big \vert \sum _{k=1}^{\infty } ( B_{s} B_{s}^{*} A_{s} ( B_{s} - B_{t} ) B_{t}^{*} A_{t} B_{t} )_{n,-k} \sqrt{k}\, h_{k} \Big \vert ^{2} , \\ I\!V_{5}&= \sum _{n=1}^{\infty } \Big \vert \sum _{k=1}^{\infty } ( B_{s} B_{s}^{*} A_{s} ( B_{t} - B_{s} ) )_{n,-k} \sqrt{k}\, h_{k} \Big \vert ^{2} . \end{aligned} \end{aligned}$$

By using Corollaries 4.8 and 4.11, it is easy to see that

$$\begin{aligned} I\!V_{i} \leqslant c_{5i} \omega (s,t)^{2} \Vert h \Vert _{ H^{1/2} }^{2} \quad \text {for }i=1,2,3,4,5, \end{aligned}$$

for some constants $c_{51}, c_{52}, c_{53}, c_{54}, c_{55} > 0$. Therefore we get

$$\begin{aligned} I\!V \leqslant c_{5} \omega (s,t)^{2} \Vert h \Vert _{ H^{1/2} }^{2} \end{aligned}$$

for some constant $c_{5} > 0$.

$$\underline{{ Estimate\, of\, V.}}$$

$$\begin{aligned} \begin{aligned} V&= \sum _{n=1}^{\infty } \Big \vert \sum _{k=1}^{\infty } ( B_{t} B_{t}^{*} A_{t} - B_{s} B_{s}^{*} A_{s} )_{n,k} \sqrt{k}\, h_{-k} \Big \vert ^{2} \end{aligned} \end{aligned}$$

Along the decomposition

$$\begin{aligned} \begin{aligned}&B_{t} B_{t}^{*} A_{t} - B_{s} B_{s}^{*} A_{s} \\&\quad = ( B_{t} - B_{s} ) B_{t}^{*} A_{t} + B_{s} ( B_{t}^{*} - B_{s}^{*} ) A_{t} + B_{s} B_{s}^{*} ( A_{t} - A_{s} ) , \end{aligned} \end{aligned}$$

the quantity *V* is estimated as

$$\begin{aligned} V \leqslant 3 ( V_{1} + V_{2} + V_{3} ), \end{aligned}$$

where

$$\begin{aligned} \begin{aligned} V_{1}&= \sum _{n=1}^{\infty } \Big \vert \sum _{k=1}^{\infty } ( ( B_{t} - B_{s} ) B_{t}^{*} A_{t} )_{n,k} \sqrt{k}\, h_{-k} \Big \vert ^{2} \\ V_{2}&= \sum _{n=1}^{\infty } \Big \vert \sum _{k=1}^{\infty } ( B_{s} ( B_{t}^{*} - B_{s}^{*} ) A_{t} )_{n,k} \sqrt{k}\, h_{-k} \Big \vert ^{2}, \\ V_{3}&= \sum _{n=1}^{\infty } \Big \vert \sum _{k=1}^{\infty } ( B_{s} B_{s}^{*} ( A_{t} - A_{s} ) )_{n,k} \sqrt{k}\, h_{-k} \Big \vert ^{2} . \end{aligned} \end{aligned}$$

By Corollaries 4.8 and 4.11, we have

$$\begin{aligned} \begin{aligned} V_{1}&\leqslant \Vert B_{t} - B_{s} \Vert ^{2} \Vert B_{t}^{*} A_{t} \Vert ^{2} \Vert \Lambda h \Vert _{H}^{2} \leqslant c_{51} \omega (s,t)^{2} \Vert h \Vert _{ H^{1/2} }^{2}, \\ V_{2}&\leqslant \Vert B_{s} \Vert ^{2} \Vert B_{t}^{*} - B_{s}^{*} \Vert ^{2} \Vert A_{t} \Vert ^{2} \Vert \Lambda h \Vert _{H}^{2} \leqslant c_{52} \omega (s,t)^{ 2 } \Vert h \Vert _{ H^{1/2} }^{2} , \\ V_{3}&\leqslant \Vert B_{s} B_{s}^{*} \Vert ^{2} \Vert A_{t} - A_{s} \Vert ^{2} \Vert \Lambda h \Vert _{H}^{2} \leqslant c_{53} \omega (s,t)^{ 2 } \Vert h \Vert _{ H^{1/2} }^{2} , \end{aligned} \end{aligned}$$

for some constants $c_{51}, c_{52}, c_{53} > 0$. Combining these estimates, we get

$$\begin{aligned} \begin{aligned} V \leqslant c_{5} \omega (s,t)^{ 2 } \Vert h \Vert _{ H^{1/2} }^{2} \end{aligned} \end{aligned}$$

for some constant $c_{5} > 0$.

Now by combining the estimates for *I*, $I\!\!I$, $I\!\!I\!\!I$, $I\!V$ and *V*, we obtain the assertion. $\square$

## Appendix A

### Proof of Corollary 4.6

We write $f_{t}(z)$ as $f_{t} (z) = C(t) \sum _{n=1}^{\infty } c_{n}(t) z^{n}$, and then it is enough to show that $\sup _{ z \in {\mathbb {D}} } \sum _{n=1}^{\infty } \vert c_{n} (t) z^{n} \vert < +\infty ,$ for each $t \in [0,T]$. By [1, Theorem 2.8],

$$\begin{aligned} \begin{aligned} \vert c_{n}(t) \vert \leqslant \sum _{p=1}^{n} \sum _{\begin{array}{c} i_{1}, \ldots , i_{p} \in {\mathbb {N}}: \\ i_{1} + \cdots + i_{p} = n \end{array}} {\widetilde{w}} (n)_{ i_{1}, \ldots , i_{p} } \frac{ \omega (0,t)^{n} }{ n! }, \end{aligned} \end{aligned}$$

where

$$\begin{aligned} \begin{aligned} {\widetilde{w}} (n)_{ i_{1}, \ldots , i_{p} }&= \{ ( n - i_{1} ) + 1 \} \{ ( n - ( i_{1} + i_{2} ) ) + 1 \} \cdots \{ ( n - ( i_{1} + \cdots + i_{p-1} ) ) + 1 \} \\&\leqslant \{ ( n - 1 ) + 1 \} \{ ( n - 2 ) + 1 \} \cdots \{ ( n - ( p-1 ) ) + 1 \} \\&= n (n-1) \cdots (n-p) = n \left( {\begin{array}{c}n-1\\ p\end{array}}\right) p! \leqslant n 2^{n-1} p! . \end{aligned} \end{aligned}$$

Therefore,

$$\begin{aligned} \begin{aligned} \vert c_{n}(t) \vert&\leqslant n 2^{n-1} \frac{ \omega (0,t)^{n} }{ n! } \sum _{p=1}^{n} p! \sum _{\begin{array}{c} i_{1}, \ldots , i_{p} \in {\mathbb {N}}: \\ i_{1} + \cdots + i_{p} = n \end{array}} 1 \\&= n 2^{n-1} \frac{ \omega (0,t)^{n} }{ n! } \sum _{p=1}^{n} p! \left( {\begin{array}{c}n-1\\ p-1\end{array}}\right) \\&\leqslant n 2^{n-1} \omega (0,t)^{n} \sum _{p=1}^{n} \left( {\begin{array}{c}n-1\\ p-1\end{array}}\right) \\&= n 4^{n-1} \omega (0,t)^{n} = 4^{-1} n ( 4 \omega (0,t) )^{n} . \end{aligned} \end{aligned}$$

Hence, $$ \sup _{ z \in {\mathbb {D}} } \sum _{n=1}^{\infty } \vert c_{n} (t) z^{n} \vert \leqslant \sum _{n=1}^{\infty } \vert c_{n} (t) \vert < +\infty $$ if $$\omega (0,T) < \frac{1}{4}$$.

### Proof of Corollary 4.10

(i) is immediate from Definition 3.2 since $b_{-1,-1}(t)$ is explicitly given by

$$\begin{aligned} b_{-1,-1} (t) = - \text {e}^{2x_{0} (t)} \int _{0}^{t} \text {e}^{ -2x_{0} (u) } \text {d} x_{2} (u) \end{aligned}$$

(see [1, Proposition 2.10–(ii)]).

(ii) Since the Loewner–Kufarev equation is controlled by $\omega$, we have that

(for the notation  , see [1, Definition 2.11]) if $k + ( i_{1} + \cdots + i_{p} ) + (j_{1} + \cdots + j_{q}) = n+m$. Then by [1, Theorem 2.12], we get

$$\begin{aligned} \begin{aligned}&\vert b_{-m,-n} (t) \vert \\&\quad \leqslant \sum _{k=2}^{n+m-2} \sum _{\begin{array}{c} 1 \leqslant i< m; \\ 1 \leqslant j < n: \\ i+j=k \end{array}} \sum _{p=1}^{m-i} \sum _{q=1}^{n-j} \sum _{\begin{array}{c} i_{1}, \ldots , i_{p} \in {\mathbb {N}}: \\ i_{1} + \cdots + i_{p} = m-i \end{array}} \sum _{\begin{array}{c} j_{1}, \ldots , j_{q} \in {\mathbb {N}}: \\ j_{1} + \cdots + j_{q} = n-j \end{array}} w_{i_{1},\ldots , i_{p}; j_{1}, \ldots , j_{q}} \frac{ (p+q)! }{ p! q! } \frac{\omega (0,t)^{n+m}}{(n+m)!} \\&\qquad + \sum _{k=m+1}^{n+m-1} \sum _{q=1}^{n+m-k} \sum _{\begin{array}{c} j_{1}, \ldots , j_{q} \in {\mathbb {N}}: \\ j_{1} + \cdots + j_{q} = n+m-k \end{array}} w_{ \varnothing ; j_{1}, \ldots , j_{q} } \frac{ \omega (0,t)^{n+m} }{ (n+m)! } \\&\qquad + \sum _{k=n+1}^{n+m-1} \sum _{p=1}^{m+n-k} \sum _{\begin{array}{c} i_{1}, \ldots , i_{p} \in {\mathbb {N}}: \\ i_{1} + \cdots + i_{p} = m+n-k \end{array}} w_{ i_{1}, \ldots , i_{p}; \varnothing } \frac{ \omega (0,t)^{n+m} }{ (n+m)! }, \end{aligned} \end{aligned}$$

where, in the first term on the right hand side, we have $( m - ( i_{1} + \cdots + i_{p} )) = i$, $( n - ( j_{1} + \cdots + j_{q} )) = j$ and hence

$$\begin{aligned} \begin{aligned} w_{i_{1}, \ldots , i_{p}; j_{1}, \ldots , j_{q}}&= ( m-i_{1} ) ( m- (i_{1}+i_{2}) ) \cdots ( m - (i_{1}+\cdots + i_{p}) ) \\&\qquad \times ( n-j_{1} ) ( n- (j_{1}+j_{2}) ) \cdots ( n - (j_{1}+\cdots + j_{q}) ) \\&\leqslant i (m-1) (m-2) \cdots (m-(p-1)) \\&\qquad \times j (n-1) (n-2) \cdots (n-(q-1)) \\&= ij \left( {\begin{array}{c}m-1\\ p-1\end{array}}\right) (p-1)! \left( {\begin{array}{c}n-1\\ q-1\end{array}}\right) (q-1)! . \end{aligned} \end{aligned}$$

In the second and third term, we have $( n - ( j_{1} + \cdots + j_{q} )) = k-m$, $( m - ( i_{1} + \cdots + i_{p} )) = k-n$, so that

$$\begin{aligned} \begin{aligned}&w_{ \varnothing ; j_{1}, \ldots , j_{q} } \leqslant (k-m) \left( {\begin{array}{c} n-1 \\ q-1 \end{array}}\right) (q-1)! , \\&w_{ i_{1}, \ldots , i_{q} ; \varnothing } \leqslant (k-n) \left( {\begin{array}{c} m-1 \\ p-1 \end{array}}\right) (p-1)! . \end{aligned} \end{aligned}$$

So we have

$$\begin{aligned}&\vert b_{-m,-n} (t) \vert \\&\quad \leqslant \sum _{k=2}^{n+m-2} \sum _{\begin{array}{c} 1 \leqslant i< m; \\ 1 \leqslant j< n: \\ i+j=k \end{array}} \sum _{p=1}^{m-i} \sum _{q=1}^{n-j} \sum _{\begin{array}{c} i_{1}, \ldots , i_{p} \in {\mathbb {N}}: \\ i_{1} + \cdots + i_{p} = m-i \end{array}} \sum _{\begin{array}{c} j_{1}, \ldots , j_{q} \in {\mathbb {N}}: \\ j_{1} + \cdots + j_{q} = n-j \end{array}} ij \left( {\begin{array}{c}m-1\\ p-1\end{array}}\right) \left( {\begin{array}{c}n-1\\ q-1\end{array}}\right) (p-1)! (q-1)! \\&\qquad \times \frac{ (p+q)! }{ p! q! } \frac{\omega (0,t)^{n+m}}{(n+m)!} \\&\qquad + \sum _{k=m+1}^{n+m-1} \sum _{q=1}^{n+m-k} \sum _{\begin{array}{c} j_{1}, \ldots , j_{q} \in {\mathbb {N}}: \\ j_{1} + \cdots + j_{q} = n+m-k \end{array}} (k-m) \left( {\begin{array}{c} n-1 \\ q-1 \end{array}}\right) (q-1)! \frac{ \omega (0,t)^{n+m} }{ (n+m)! } \\&\qquad + \sum _{k=n+1}^{n+m-1} \sum _{p=1}^{m+n-k} \sum _{\begin{array}{c} i_{1}, \ldots , i_{p} \in {\mathbb {N}}: \\ i_{1} + \cdots + i_{p} = m+n-k \end{array}} (k-n) \left( {\begin{array}{c} m-1 \\ p-1 \end{array}}\right) (p-1)! \frac{ \omega (0,t)^{n+m} }{ (n+m)! } \\&\quad = \sum _{k=2}^{n+m-2} \sum _{\begin{array}{c} 1 \leqslant i< m; \\ 1 \leqslant j < n: \\ i+j=k \end{array}} ij \sum _{p=1}^{m-i} \sum _{q=1}^{n-j} \Big ( \sum _{\begin{array}{c} i_{1}, \ldots , i_{p} \in {\mathbb {N}}: \\ i_{1} + \cdots + i_{p} = m-i \end{array}} 1 \Big ) \Big ( \sum _{\begin{array}{c} j_{1}, \ldots , j_{q} \in {\mathbb {N}}: \\ j_{1} + \cdots + j_{q} = n-j \end{array}} 1 \Big ) \\&\qquad \times \left( {\begin{array}{c}m-1\\ p-1\end{array}}\right) \left( {\begin{array}{c}n-1\\ q-1\end{array}}\right) (p-1)! (q-1)! \frac{ (p+q)! }{ p! q! } \frac{\omega (0,t)^{n+m}}{(n+m)!} \\&\qquad + \sum _{k=m+1}^{n+m-1} \sum _{q=1}^{n+m-k} \Big ( \sum _{\begin{array}{c} j_{1}, \ldots , j_{q} \in {\mathbb {N}}: \\ j_{1} + \cdots + j_{q} = n+m-k \end{array}} 1 \Big ) (k-m) \left( {\begin{array}{c} n-1 \\ q-1 \end{array}}\right) (q-1)! \frac{ \omega (0,t)^{n+m} }{ (n+m)! } \\&\qquad + \sum _{k=n+1}^{n+m-1} \sum _{p=1}^{m+n-k} \Big ( \sum _{\begin{array}{c} i_{1}, \ldots , i_{p} \in {\mathbb {N}}: \\ i_{1} + \cdots + i_{p} = m+n-k \end{array}} 1 \Big ) (k-n) \left( {\begin{array}{c} m-1 \\ p-1 \end{array}}\right) (p-1)! \frac{ \omega (0,t)^{n+m} }{ (n+m)! } \end{aligned}$$

In the first term of the last equation, we have $\frac{ (p+q)! }{ p! q! } \leqslant 2^{p+q} \leqslant 2^{m+n-2}$, $(p-1)! (q-1)! \leqslant (p+q-1)! \leqslant (m+n-3)!$, and

$$\begin{aligned} \sum _{\begin{array}{c} i_{1}, \ldots , i_{p} \in {\mathbb {N}}: \\ i_{1} + \cdots + i_{p} = m-i \end{array}} 1 = \left( {\begin{array}{c} m-i-1 \\ p-1 \end{array}}\right) , \quad \sum _{\begin{array}{c} j_{1}, \ldots , j_{q} \in {\mathbb {N}}: \\ j_{1} + \cdots + j_{q} = n-j \end{array}} 1 = \left( {\begin{array}{c} n-j-1 \\ q-1 \end{array}}\right) . \end{aligned}$$

For the second and the third terms, we have $(q-1)! \leqslant (n+m-3)!$ and $(p-1)! \leqslant (n+m-3)!$ respectively. Then we obtain

$$\begin{aligned}&\vert b_{-m,-n} (t) \vert \\&\quad \leqslant \omega (0,t)^{n+m} \frac{ (m+n-3)! 2^{m+n-2} }{ (n+m)! } \Big \{ \sum _{k=2}^{n+m-2} \sum _{\begin{array}{c} 1 \leqslant i< m; \\ 1 \leqslant j < n: \\ i+j=k \end{array}} ij \\&\qquad \times \Big ( \sum _{p^{\prime }=0}^{m-i-1} \left( {\begin{array}{c} m-1 \\ p^{\prime } \end{array}}\right) \left( {\begin{array}{c} m-i-1 \\ (m-i-1) - p^{\prime } \end{array}}\right) \Big ) \Big ( \sum _{ q^{\prime } = 0 }^{ n-j-1 } \left( {\begin{array}{c} n-1 \\ q^{\prime } \end{array}}\right) \left( {\begin{array}{c} n-j-1 \\ (n-j-1) - q^{\prime } \end{array}}\right) \Big ) \\&\qquad + \sum _{k=m+1}^{n+m-1} (k-m) \sum _{q=1}^{n+m-k} \left( {\begin{array}{c} n+m-k-1 \\ q-1 \end{array}}\right) \left( {\begin{array}{c} n-1 \\ q-1 \end{array}}\right) \\&\qquad + \sum _{k=n+1}^{n+m-1} (k-n) \sum _{p=1}^{m+n-k} \left( {\begin{array}{c} n+m-k-1 \\ p-1 \end{array}}\right) \left( {\begin{array}{c} m-1 \\ p-1 \end{array}}\right) \Big \} . \end{aligned}$$

The combinatorial formulae

$$\begin{aligned} \sum _{p^{\prime }=0}^{m-i-1} \left( {\begin{array}{c} m-1 \\ p^{\prime } \end{array}}\right) \left( {\begin{array}{c} m-i-1 \\ (m-i-1) - p^{\prime } \end{array}}\right)&= \left( {\begin{array}{c} 2(m-1)-i \\ m-1 \end{array}}\right) , \\ \sum _{ q^{\prime } = 0 }^{n-j-1} \left( {\begin{array}{c} n-1 \\ q^{\prime } \end{array}}\right) \left( {\begin{array}{c} n-j-1 \\ (n-j-1) - q^{\prime } \end{array}}\right)&= \left( {\begin{array}{c} 2(n-1)-j \\ n-1 \end{array}}\right) \end{aligned}$$

give

$$\begin{aligned}&\vert b_{-m,-n} (t) \vert \\&\quad \leqslant \omega (0,t)^{n+m} \frac{ (m+n-3)! 2^{m+n-2} }{ (n+m)! } \Big \{ \sum _{k=2}^{n+m-2} \sum _{\begin{array}{c} 1 \leqslant i< m; \\ 1 \leqslant j < n: \\ i+j=k \end{array}} ij \left( {\begin{array}{c} 2(m-1)-i \\ m-1 \end{array}}\right) \left( {\begin{array}{c} 2(n-1)-j \\ n-1 \end{array}}\right) \\&\qquad + \sum _{k=m+1}^{n+m-1} (k-m) \left( {\begin{array}{c} 2(n-1) - (k-m) \\ n-1 \end{array}}\right) \\&\qquad + \sum _{k=n+1}^{n+m-1} (k-n) \left( {\begin{array}{c} 2(m-1) - (k-n) \\ m-1 \end{array}}\right) \Big \} \\&\quad = \omega (0,t)^{n+m} \frac{ (m+n-3)! 2^{m+n-2} }{ (n+m)! } \Big \{ \Big ( \sum _{ i = 1 }^{ m-1 } i \left( {\begin{array}{c} 2(m-1)-i \\ m-1 \end{array}}\right) \Big ) \Big ( \sum _{ j = 1 }^{ n-1 } j \left( {\begin{array}{c} 2(n-1)-j \\ n-1 \end{array}}\right) \Big ) \\&\qquad + \sum _{k=1}^{n-1} k \left( {\begin{array}{c} 2(n-1) - k \\ n-1 \end{array}}\right) + \sum _{k=1}^{m-1} (k-n) \left( {\begin{array}{c} 2(m-1) - k \\ m-1 \end{array}}\right) \Big \} . \end{aligned}$$

We shall use here the identity $$ \sum _{j=k}^{n} ( n+1-j ) \left( {\begin{array}{c} j-1 \\ k-1 \end{array}}\right) = \left( {\begin{array}{c} n+1 \\ k+1 \end{array}}\right) $$ which enables us to conclude

$$\begin{aligned} \begin{aligned} \sum _{ i = 1 }^{ m-1 } i \left( {\begin{array}{c} 2(m-1)-i \\ m-1 \end{array}}\right) = \sum _{j=m}^{2(m-1)} ( 2(m-1) + 1 - j ) \left( {\begin{array}{c} j-1 \\ m-1 \end{array}}\right) = \left( {\begin{array}{c}2m-1\\ m\end{array}}\right) . \end{aligned} \end{aligned}$$

Hence, we conclude that

$$\begin{aligned}&\vert b_{-m,-n} (t) \vert \\&\quad \leqslant \omega (0,t)^{m+n} \frac{ (m+n-3)! 2^{m+n-2} }{ (m+n)! } \Big \{ \left( {\begin{array}{c}2m-1\\ m\end{array}}\right) \left( {\begin{array}{c}2n-1\\ n\end{array}}\right) + \left( {\begin{array}{c}2n-1\\ n\end{array}}\right) + \left( {\begin{array}{c}2m-1\\ m\end{array}}\right) \Big \} \\&\quad \leqslant \omega (0,t)^{m+n} \frac{ (m+n-3)! 2^{m+n-2} }{ (m+n)! } \Big \{ \left( {\begin{array}{c}2m-1\\ m\end{array}}\right) + 1 \Big \} \Big \{ \left( {\begin{array}{c}2n-1\\ n\end{array}}\right) + 1 \Big \} \\&\quad \leqslant \frac{ \omega (0,t)^{m+n} 2^{m+n-2} ( 2^{2m-1} 2^{2n-1} ) }{ (m+n) (m+n-1) (m+n-2) } = \frac{ (8\omega (0,t))^{m+n} }{ 16 (m+n) (m+n-1) (m+n-2) }. \end{aligned}$$

(iii) Since the Loewner–Kufarev equation is controlled by $\omega$, we have

Now the remaining case is the same as (i).

## Footnotes

1 Here we avoid the conflicting use of the word “controlled”, which has two meanings, cf. [12] p. 16.
